# Assessment of acute radial artery injury after distal transradial access for coronary intervention: an optical coherence tomography study

**DOI:** 10.1007/s00380-024-02461-y

**Published:** 2024-09-25

**Authors:** Dan Niu, Yuntao Wang, Yongxia Wu, Zixuan Li, Hao Liu, Jincheng Guo

**Affiliations:** 1 Department of Cardiology, Affiliated Hospital of Inner Mongolia Medical University, Hohhot, Inner Mongolia China; 2 Department of Cardiology, Beijing Luhe Hospital, Capital Medical University, Beijing, China

**Keywords:** Distal transradial access, Radial artery, Optical coherence tomography

## Abstract

There is a paucity of data on acute radial artery (RA) injuries using optical coherence tomography (OCT) in patients undergoing coronary intervention via distal transradial coronary access (dTRA). To evaluate the incidence of acute RA injury following dTRA for coronary intervention using OCT. We retrospectively analyzed 200 consecutive patients with acute coronary syndrome (ACS) who underwent coronary intervention guided by OCT and RA-OCT after dTRA at our center between June 2021 and November 2022. Total length of RA was divided into three segments based on the sheath location during dTRA: no sheath protection portion (proximal RA segment) and sheath protection portion (divided into mid- and distal segments). Acute RA injuries, including tears, dissections, perforations, thrombi, and spasms, were analyzed. Radial artery occlusion (RAO) was assessed using ultrasonography 24 h after dTRA. Acute RA injury was observed in 45.5% of patients after dTRA. The incidence of tear, dissection, perforation, thrombi, and spasm in all the patients was 11.5%, 16.5%, 1.5%, 17.5%, and 17.5%, respectively. In segment-level analysis, dissection and spasm were significantly more frequent in the proximal segment, followed by the mid and distal segments (11.0% vs. 5.5% vs. 4.5%, *P* = 0.015; 13.0% vs. 4.0% vs. 4.5%, *P* = 0.002). The rate of RAO at 24-h follow-up was 3.0%. Acute RA injuries were observed in nearly half of the patients using OCT via dTRA; dissection and spasm occurred more frequently in the proximal segment. Hydrophilic-coated sheaths have the potential advantage of preventing radial artery spasm and dissection.

**Supplementary Information:**

The online version contains supplementary material available at 10.1007/s00380-024-02461-y.

## Introduction

Radial access is recommended as the standard approach for coronary angiography and percutaneous coronary intervention (PCI) [1]. Although transradial access is generally considered safe, the insertion of an introducer sheath, guidewire, or guide catheter manipulation may cause acute radial artery (RA) injuries, such as RA spasm, dissection, perforation, thrombus, or occlusion, restricting the ability to use the same access for future coronary interventions, coronary artery bypass grafting, or an arteriovenous fistula for dialysis [2, 3].

High-resolution optical coherence tomography (OCT) is the most accurate method to measure RA injury. Previous studies have arrived at differing conclusions regarding the role of introducer sheath length in reducing acute RA injury during transradial access (TRA). The difference in the acute RA-injury pattern between the segment with no sheath protection and the segment with sheath protection remains unclear.

Distal transradial coronary access (dTRA) in the anatomical snuffbox has received much attention in recent years because of its physiological and anatomic advantages over TRA, which offer potentially faster access site haemostasis and lower rates of radial artery occlusion (RAO) [4]. However, limited data are available regarding the incidence of RA-injury patterns using dTRA.

This study aimed to observe and compare the incidence of acute RA-injury patterns and distribution post-PCI via dTRA using frequency-domain OCT.

## Methods

### Patient selection

This was a retrospective study involving ACS patients enrolled at a single center (Beijing Luhe Hospital) between June 2021 and November 2022. We screened patients presenting with ACS and undergoing RA-OCT via dTRA after the completion of OCT-guided coronary intervention (Supplementary Figure). Among 397 patients, 187 patients with a prior history of ipsilateral forearm radial or distal radial access, and 10 patients with poor-quality OCT images were excluded. Consequently, 200 patients were included in this analysis. This study was approved by the Institutional Review Board of Beijing Luhe Hospital (No. 2024-LHKY-025-02), and all patients provided written informed consent before the cardiac catheterization procedures.

### PCI and OCT procedures

A puncture was performed at the snuffbox of the distal RA using the Seldinger technique. Subsequently, a 0.025-inch guidewire was inserted, followed by the introduction of a 6F conventional sheath with a length of 16 cm (Terumo Co., Tokyo, Japan). The use of unfractionated heparin (UFH) or bivalirudin, and the choice of the platelet glycoprotein IIb/IIIa receptor antagonist (tirofiban), were at the physician’s discretion. Typically, a 5F TIG multifunctional angiography catheter (Terumo. Corporation, Japan) was used for the angiographic process, while a 6F guiding catheter was used for the PCI procedure. Thrombus aspiration, balloon dilation, and stent placement depended on the operators’ decisions.

**Fig. 1:**
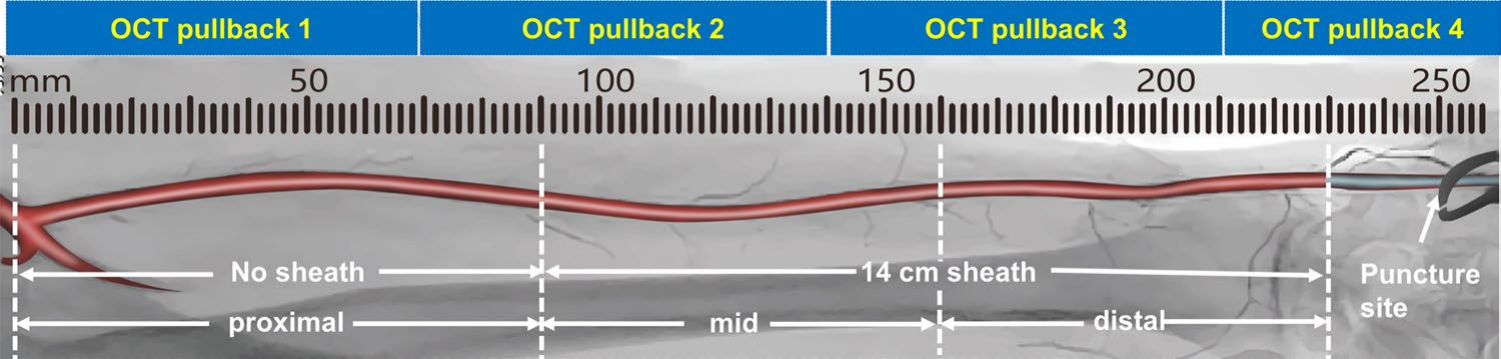
Illustration of OCT analysis of the radial artery. Radial artery angiogram was performed with a radiopaque ruler placed at the radial artery ostium; a pair of Pean forceps is located at the puncture site. The entire image length starts from radial artery ostium and ends at 2 cm distal to the puncture site. Three or four OCT pullbacks were performed with 2 or 3 overlaps based on the whole radial artery length. Acute injuries were recorded according to the following three segments: distal—centre of the sheath to 2 cm distal to the puncture site; mid—tip of the sheath to the centre of the sheath; proximal—radial artery ostium to the tip of the sheath

After the PCI procedure, RA angiography was performed to locate the ostium of the RA. A radiolucent X-ray ruler was placed parallel to the RA. RA-OCT imaging was performed using the commercially available OPTIS Mobile System (Abbott Vascular, Santa Clara, CA, USA) with Dragonfly OPTIS imaging catheters (Abbott Vascular). The distal lens marker of the OCT imaging catheter was placed at the ostium of the RA. After injection of 0.2 mg nitroglycerin and verapamil (2.5 mg), the sheath was pulled back to a position 2 cm proximal to the puncture site. Subsequently, a 50-ml syringe filled with normal saline was connected to the sheath side port and rapidly flushed to clear blood away, ensuring a clear OCT image was obtained. Immediately thereafter, the OCT imaging catheter was pulled back with an automatic speed of 20 mm/s, from ostium to sheath tip, with 3 or 4 runs. Figure 1 shows the operation of OCT in the RA. At the end of the RA-OCT examination, haemostasis was achieved using a radial compression device (Air Power, Shenzhen, China) for 3 h. The incidence of RAO 24 h after dTRA was also measured using Doppler ultrasonography. All procedures were performed by two experienced interventional physicians.

### OCT image evaluation

**Fig. 2:**
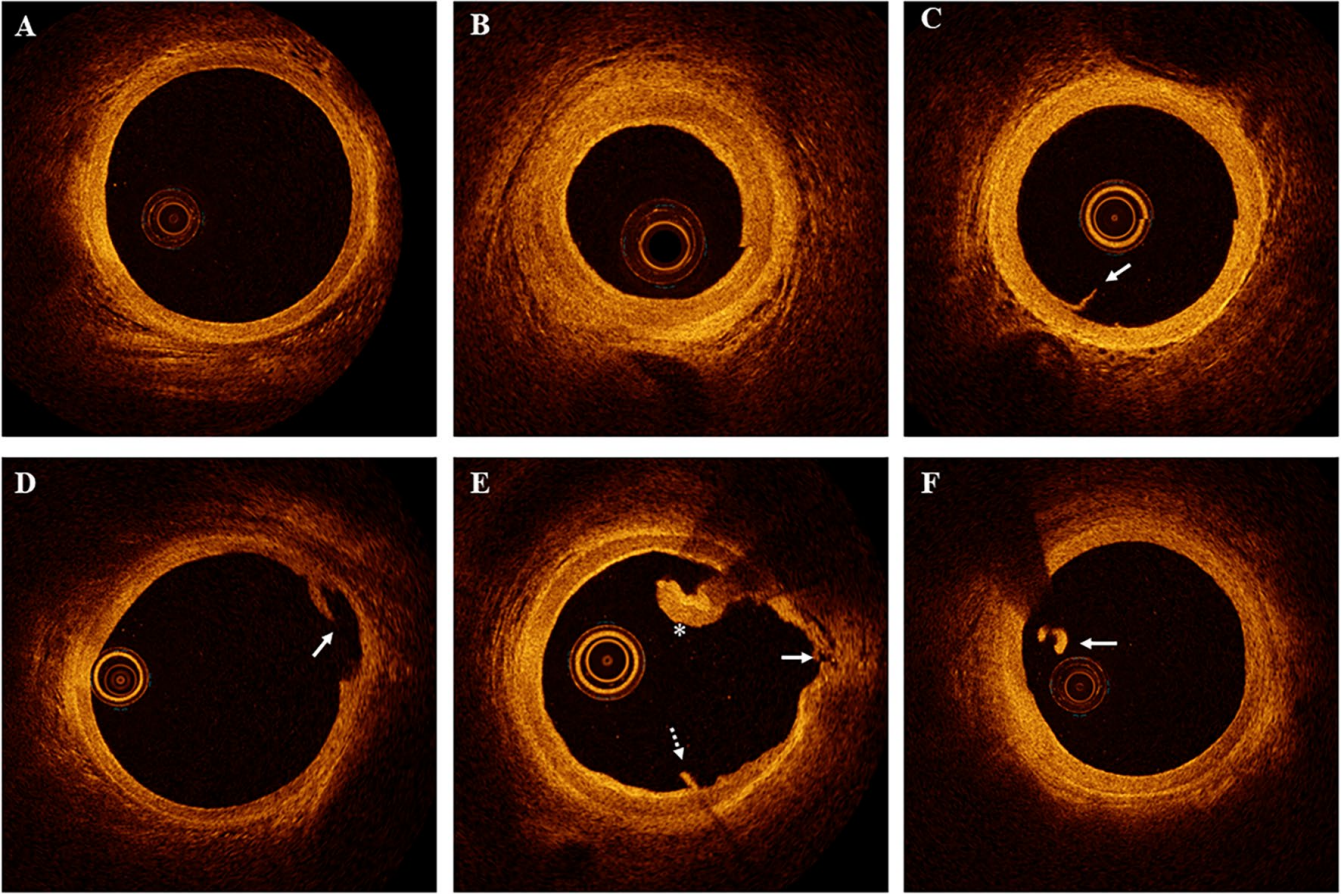
Typical optical coherence tomography images of radial artery injuries. **A** Normal radial artery structure. **B** Spasm: apparent decreasing of vessel area and increasing of thickness of the medial layer. **C** Intimal tear: intimal layer discontinuity at 7–8 o’ clock (arrow). **D** Dissection: medial layer discontinuity at 2–3 o’ clock (arrow). **E** Perforation: complete loss of vessel wall continuity with fluid leak into the interstitial tissue space at 3 o’ clock (arrow) and with intimal tear (dot arrow) at 5–6 o’ clock and thrombus at 1–2 o’ clock (asterisk). **F** Thrombus: a mass attached to the luminal surface or floating in the lumen (arrow)

All OCT images were reviewed by two independent investigators, who were blinded to the clinical, angiographical and laboratory data, using an offline review workstation (Abbott Vascular). Any discordance between the two observers were resolved through consensus with a third reviewer. The entire length of the RA was divided into the proximal segment with no sheath protection (from the RA ostium to the tip of the sheath), the mid-segment (from the tip of the sheath to the center of the sheath, 7 cm length), and the distal segment (from the center of the sheath to 2 cm distal to the puncture site, 7 cm length) with the sheath present. Acute injuries, including intimal tears, dissection, perforation, thrombi, and spasms were recorded, and the definitions were based on established criteria [5–7]. An intimal tear was defined as a luminal surface discontinuity restricted to the intimal layer. Dissection refers to the luminal surface disruption that extends into the medial layer. Perforation refers to the complete loss of vessel wall continuity, with fluid leakage into the interstitial tissue space. A thrombus is a mass attached to the luminal surface or floating in the lumen. Sequential OCT was performed to determine the existence of spasm that was defined as a decreased change of vessel area (>50%) with an increased thickness of the medial layer (>20%) [6]. For typical cases see Fig. 2.

### Statistical analysis

The main statistical indicators were tested for normality, and the measurement data of normal distribution were represented by mean ± SD, median (interquartile range) for non-normal distribution measurement data, and *n* (%) for counting data. A comparison of the incidence of acute injuries and their mean diameters across the proximal, mid, and distal segments was conducted using nonparametric tests and paired sample *t* tests. Bilateral *P* < 0.05 indicates a statistically significant difference. All statistical analyses were conducted using SPSS software (version 23.0; IBM SPSS Statistics, Armonk, NY, USA).

## Results

In the 200 patients who enrolled in this study, the mean age was 60.3 years, and 81.0% were male. PCI was performed in 183 patients (91.5%) and coronary angiography in 17 patients (8.5%). Table 1 summarizes the clinical and procedural characteristics of the study population.

**Table 1:** Baseline characteristics of the study population

	Study population (*n* = 200)
Sex (male/female)	162/38
Age (years)	60.30 ± 14.37
LVEF (%)	66.0 (56.0, 72.0)
Scr (μmol/L)	79.0 (69.5, 91.5)
TG (mmol/L)	1.4 (1.0, 1.9)
TC (mmol/L)	4.3 (3.7, 4.9)
LDL (mmol/L)	2.8 (2.2, 3.3)
HbA1c (%)	6.0 (5.6, 7.0)
Medical history *n* (%)	
Hypertension	129 (64.5)
Diabetes	69 (34.5)
Hyperlipidemia	183 (91.5)
Smoking	112 (56)
Renal insufficiency	30 (15)
Peripheral vascular disease	8 (4)
Heart failure	37 (18.5)
CAD family history	36 (18)
Stroke	19 (9.5)
Aspirin use	9 (4.5)
Statin use	17 (8.5)
Diagnose	
STEMI	122 (61)
NSTEMI	46 (23)
UAP	32 (16)
*Intervention data*	
Number of guide wire	1.17 ± 0.44
Number of guide catheter	1.55 ± 0.92
Procedure time (min)	86.0 (67.0, 100.0)
CAG *n* (%)	17 (8.5)
PCI *n* (%)	183 (91.5)
Number of stents	1.20 ± 1.06
Anticoagulants *n* (%)	
UFH	136 (68)
Bivalirudin	64 (32)

*LVEF* left ventricular ejection fraction, *Scr* serum creatinine, *TG* triglyceride, *TC* total cholesterol, *LDL* low-density lipoprotein, *HbA1c* hemoglobin A1C, *CAD* coronary artery disease, *STEMI* ST-segment elevation myocardial infarction, *NSTEMI* non-ST-segment elevation myocardial infarction, *UAP* unstable angina pectoris, *CAG* coronary angiography, *PCI* percutaneous coronary intervention, *UFH* unfractionated heparin

The mean length of cross sections of OCT analyzed RA was 215.1 ± 16.8 mm, and the mean length of proximal RA was 75.1 ± 16.8 mm. The mean lumen diameter of RA was 3.07 ± 0.47 mm. There was no difference between the proximal (3.08 ± 0.48 mm), mid (3.07 ± 0.61 mm), and distal (3.05 ± 0.44 mm) segments of RA in mean lumen diameter (*P* = 0.146).

**Fig. 3:**
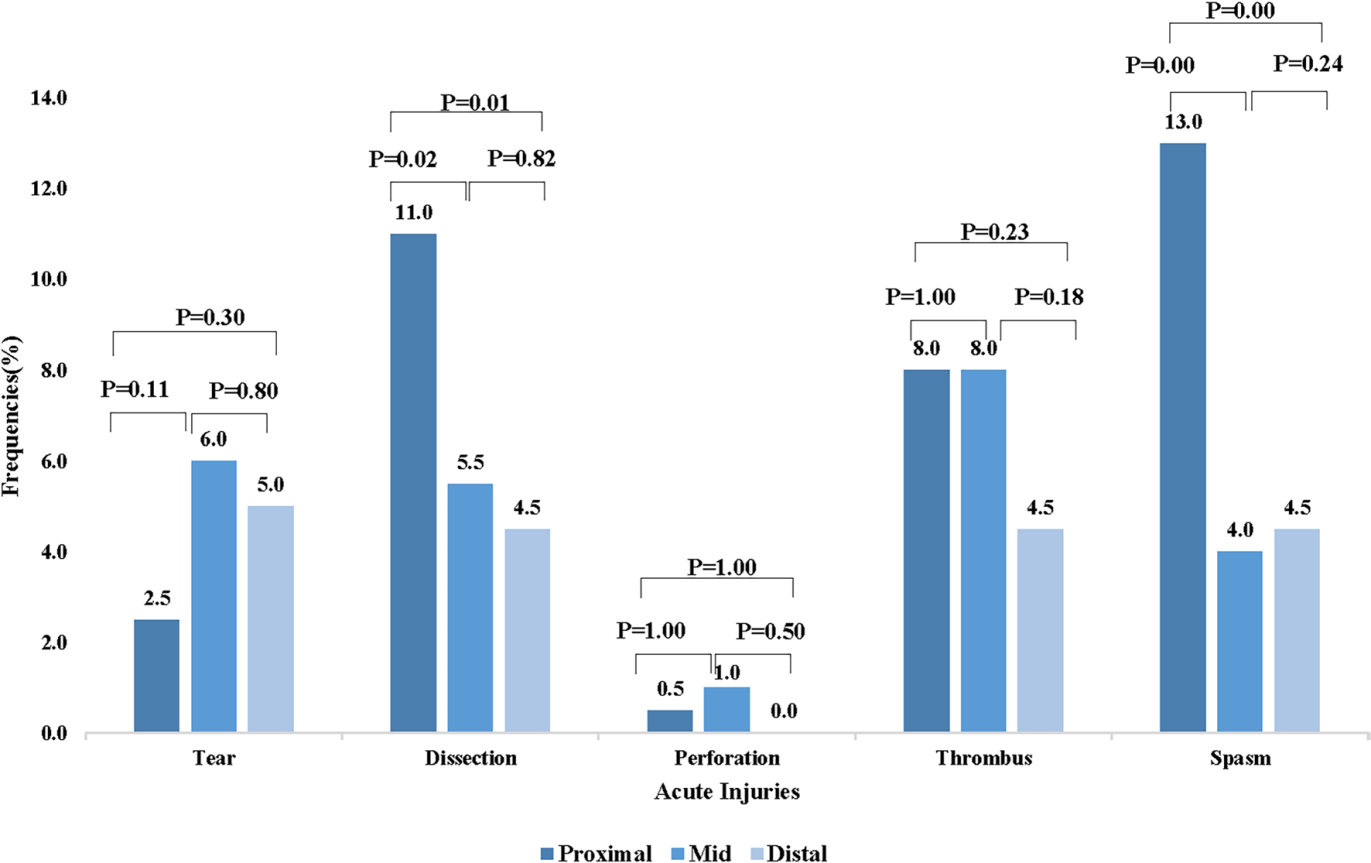
Frequencies and comparison of acute injuries in each segment of the RA. Frequencies of intimal tears, medial dissections, perforation, thrombus and spasm. The frequency of dissection and spasm was significantly higher in the proximal than in the mid- and distal segment

Acute radial injuries were assessed across 600 segments of 200 RAs. A total of 91 patients (45.5%) were observed to have acute injuries, with occurrences of intimal tears, dissections, perforations, thrombi, and spasms detected at rates of 11.5%, 16.5%, 1.5%, 17.5%, and 17.5%, respectively. Acute RA injuries occurred significantly more often in the proximal RA (29.0%) compared to the mid (19.5%) and distal RA (15.0%) segments, *P* < 0.001. Dissections and spasms were more frequently observed in the proximal RA compared to the mid and distal RA segments (for dissections: 11.0% vs. 5.5% vs. 4.5%, *P* = 0.012; for spasms: 13.0% vs. 4.0% vs. 4.5%, *P* < 0.001). No significant differences were observed in the frequency of tears, thrombi, and perforations in the proximal, mid, and distal RA segments. Table 2 and Fig. 3 illustrate the frequencies and comparisons of acute radial injuries across each RA segment. Ultrasound examinations revealed that six patients (3%) developed RAO 24 h post-PCI.

**Table 2:** Qualitative OCT measurements between the three segments

Parameters	Whole RA	Proximal	Mid	Distal	*P* value
Number of totally analyzed cross sections (frame)	1077.5 ± 84.0	375.5 ± 84.0	350	350	0.804
Length of analyzed RA (mm)	215.1 ± 16.8	75.1 ± 16.8	70	70	0.804
Lumen diameter of RA (mm)	3.07 ± 0.47	3.08 ± 0.48	3.07 ± 0.61	3.05 ± 0.44	0.146
Acute injury of RA via dTRI					
Total acute injury (%)	45.5	29.0	19.5	15.0	0.000
Length of total acute injury (mm)	6.0 (3.0, 14.0)	5.5 (2.0, 11.0)	3.0 (2.0, 7.0)	5.0 (2.0, 10.0)	0.002
Tear (%)	11.5	2.5	6.0	5.0	0.183
Length of tear (mm)	3.0 (1.0, 10.0)	4.0 (2.0, 8.0)	3.0 (1.0, 6.5)	3.0 (1.0, 8.5)	0.162
Dissection (%)	16.5	11.0	5.5	4.5	0.012
Length of dissection (mm)	5.0 (2.0, 11.5)	5.5 (2.0, 8.8)	5.0 (2.0, 9.0)	2.0 (2.0, 10.5)	0.042
Perforation (%)	1.5	0.5	1.0	0	0.368
Length of perforation (mm)	1.0 (1.0, 1.0)	1.0 (1.0, 1.0)	1.0 (1.0, 1.0)	0	0.368
Thrombus (%)	17.5	8.0	8.0	4.5	0.247
Length of thrombus (mm)	2.0 (1.0, 3.0)	2.0 (1.0, 3.8)	2.0 (1.0, 4.0)	2.0 (2.0, 6.0)	0.260
Spasm (%)	17.5	13.0	4.0	4.5	0.000
Length of spasm (mm)	8.0 (5.0, 16.0)	6.0 (4.8, 21.3)	4.5 (2.0, 10.8)	8.0 (5.0, 10.5)	0.001

Values are expressed as mean ± standard deviation, median (interquartile range), or *n* (%)

*OCT* optical coherence tomography, *RA* radial artery, *dTRI* distal transradial intervention

## Discussion

In the present study, dTRA was associated with acute RA injury in about half of the patients, as determined by OCT. The proximal segment, where no sheath was present, exhibited significantly more dissections and spasms than the mid and distal segments, which were sheathed. No significant differences were observed in the frequency or localization of intimal tears, perforations, and thrombi among the proximal, mid, and distal segments. These findings indicate the potential for sheath protection of the RA via dTRA.

Acute RA injury may be caused by RA puncture, sheath introduction, sheath friction from the RA inner diameter-sheath outer diameter mismatch or catheter, advancement of standard or hydrophilic guidewires, diagnostic catheters, or guiding catheters [8]. Currently, imaging methods used to clinically evaluate the structure of the RA include non-invasive high-frequency Doppler ultrasound, ultrasound biomicroscopy (UBM), invasive intravascular ultrasound (IVUS), RA angiography, and OCT. Non-invasive high-frequency Doppler ultrasound (spatial resolution 30–50 µm) and ultrasound biomicroscopy (UBM) (spatial resolution <70 µm) can display the rough outlines of blood vessels. Although these methods have certain advantages in evaluating injuries such as radial artery occlusion (RAO) or pseudoaneurysms, they are unable to clearly display the radial artery wall and the intraluminal structures [9, 10]. IVUS can assess the intraluminal structure of the RA and acute injuries; however, its resolution is limited to only 100 µm, which is considered low [11]. Meanwhile, OCT with a high resolution of 10–20 µm, can provide clear images of vascular walls and lumen structures. It is also more sensitive in detecting structural damage to the RA.

Determining the incidence of acute RA-injury post-procedure is challenging and varies due to factors including the observation period of the RA, sheath type and length, prophylactic vasodilatory spasmolytic therapy use, sample size, and access techniques. Reports from multiple studies indicate variances in the incidence of intimal tears (8.0–43.8%), dissections (3.0–35.6%), thromboses (0.0–24.2%), and spasms, with rates as high as 45.1% incidence detected by OCT following transradial intervention (TRI) [5–8, 12, 13]. In our study, the incidence rates of tear, dissection, thrombi, and spasm among all patients were 11.5%, 16.5%, 17.5%, and 17.5%, respectively, aligning with the previously mentioned reports.

Prior research using RA-OCT examination has demonstrated that intimal tears were observed more frequently in the distal portion with sheath protection than in the proximal portion with no sheath protection (43.8% vs. 17.8%, *P* < 0.001). Medial dissection frequencies were comparable between the distal and proximal portions (23.3% vs. 20.5%, *P* > 0.05) after TRI on time domain-OCT using a 16-cm long sheath, highlighting no benefit of sheath protection for the RA [5]. In the present study, we employed the same type and length of sheath and adhered to the same RA segmentation model with three segments outlined by Yonetsu et al. [5] yet reached different conclusions. We observed a lower rate of intimal tears that was not significantly different (5.0% in distal vs. 2.5% in proximal, *P* > 0.05), with dissection more frequent in the proximal segment (4.5% in distal vs. 11.0% in proximal, *P* < 0.05), suggesting a protective role for the sheath in preventing the razor effect of the guiding catheter during distal TRI (dTRI). These differences could be attributed to variations in sample size (42 vs. 200), the type of acute RA injury (with only tears and dissections noted in the prior study), access method (RA access vs. dTRA), vasodilators (isosorbide dinitrate vs. nitroglycerin plus verapamil), and OCT technology (first generation time-domain vs. second generation frequency-domain).

In another study, Di Vito et al. [6] used a 25-cm-long hydrophilic coated introducer sheath to cover the entire length of the RA, dividing it into proximal and distal segments. No significant differences were found in the incidence of RA acute injuries (intimal tear, medial dissection, thrombus, and spasm) between these segments, indicating that such sheaths could protect the RA against damage. Consistent with previous findings, our study revealed similar acute RA injuries within the sheath-protected segments (specifically, the mid and distal segments).

TRA causes significant structural and functional damage to the RA in situ, potentially leading to the severe complication of RAO. Injuries to RA have an important impact on the long-term patency of the RA conduit and arteriovenous fistula creation in patients requiring haemodialysis [14]. One strategy to lower the incidence of RAO is the use of dTRA. Several randomized controlled trials and meta-analyses have shown that dTRA is associated with a substantial reduction in the incidence of RAO [15–17]. In our study, RAO was diagnosed in six patients (3.0%) by ultrasonography 24 h after dTRA, aligning with previous studies yet significantly lower than the reported incidence of 7.7% of RAO within 24 h in TRA [14].

To the best of our knowledge, this is the first study to assess the incidence of acute injury throughout the entire length of the RA following a coronary procedure via dTRA using OCT. Our results provide compelling evidence that dissection and spasm are more frequently detected in the proximal RA (the sheathless part), indicating that the sheath acts as a protective factor in reducing such injuries.

## Conclusions

OCT detected a higher incidence of acute RA injury after PCI with the dTRA than previously thought. Acute injuries, including dissection and spasm, were more likely to occur in the proximal RA, where sheath protection was absent. The application of a long hydrophilic coated sheath via dTRA would be an effective means of reducing acute RA injury.

## Supplementary Information

Below is the link to the electronic supplementary material.

Supplementary file1 (TIF 8821 KB)

## Abbreviations

ACS: Acute coronary syndrome
dTRA: Distal transradial coronary access
OCT: Optical coherence tomography
PCI: Percutaneous coronary intervention
RA: Radial artery
RAO: Radial artery occlusion
TRA: Transradial access
