# Supplementary figures and images for: Assessment of acute radial artery injury after distal transradial access for coronary intervention: an optical coherence tomography study
Source: Heart Vessels. 2024 Sep 25;40(3):203–9. doi: 10.1007/s00380-024-02461-y (PMC11846716; doi:10.1007/s00380-024-02461-y)

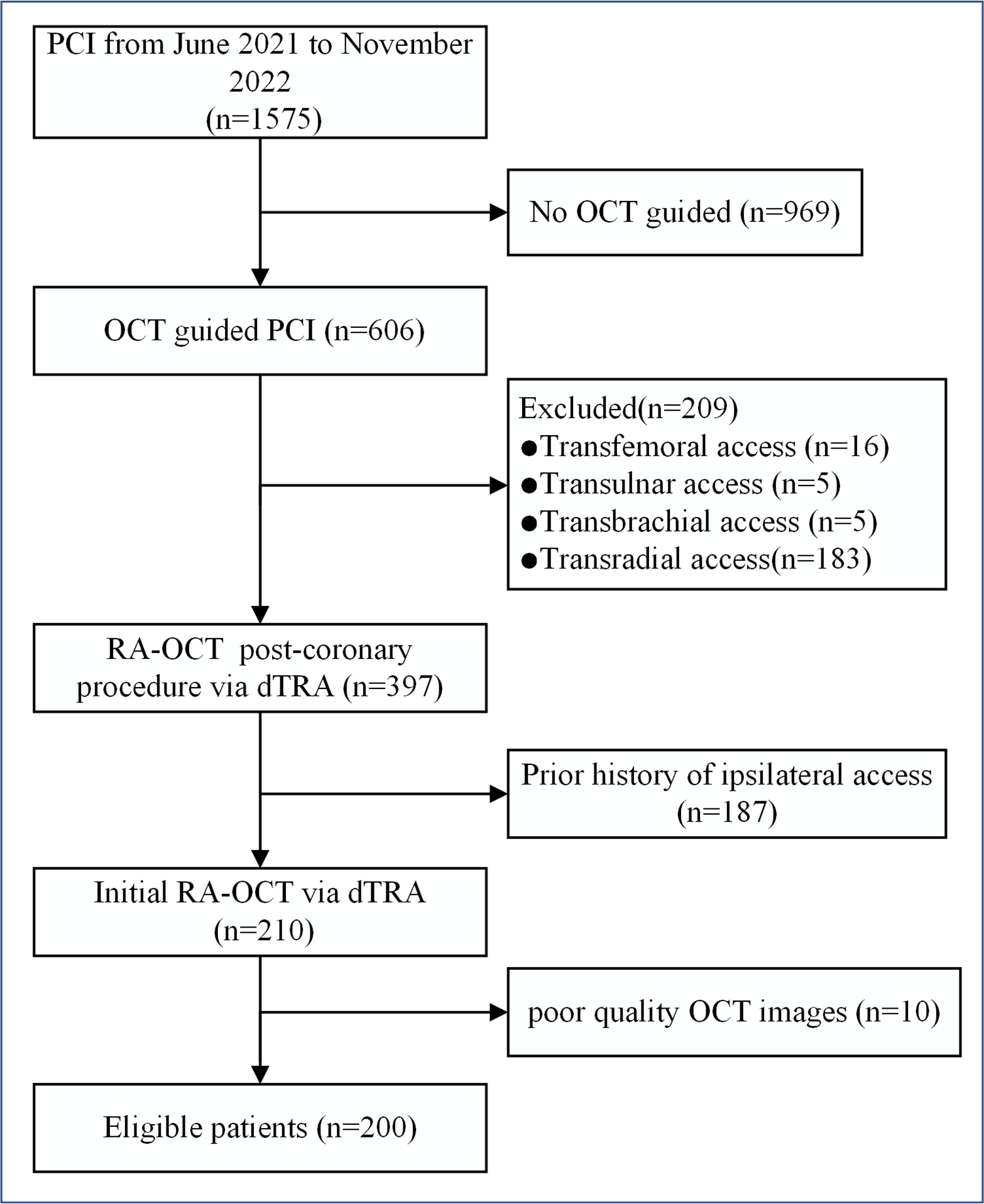

Supplement: Supplementary file 1 — Supplementary file1 (TIF 8821 KB) [file 380_2024_2461_MOESM1_ESM.tif]
